# Dual potential of microalgae as a sustainable biofuel feedstock and animal feed

**DOI:** 10.1186/2049-1891-4-53

**Published:** 2013-12-21

**Authors:** Krystal K Lum, Jonggun Kim, Xin Gen Lei

**Affiliations:** 1 Department of Animal Science, Cornell University, Ithaca, NY 14850, USA

**Keywords:** Algae, Biofuel, Feed, Health, Nutrition

## Abstract

The rise in global population has led to explorations of alternative sources of energy and food. Because corn and soybean are staple food crops for humans, their common use as the main source of dietary energy and protein for food-producing animals directly competes with their allocation for human consumption. Alternatively, de-fatted marine microalgal biomass generated from the potential biofuel production may be a viable replacement of corn and soybean meal due to their high levels of protein, relatively well-balanced amino acid profiles, and rich contents of minerals and vitamins, along with unique bioactive compounds. Although the full-fatted (intact) microalgae represent the main source of omega-3 (n-3) polyunsaturated fatty acids including docohexaenoic acid (DHA) and eicosapentaenoic acid (EPA), the de-fatted microalgal biomass may still contain good amounts of these components for enriching DHA/EPA in eggs, meats, and milk. This review is written to highlight the necessity and potential of using the de-fatted microalgal biomass as a new generation of animal feed in helping address the global energy, food, and environmental issues. Nutritional feasibility and limitation of the biomass as the new feed ingredient for simple-stomached species are elaborated. Potential applications of the biomass for generating value-added animal products are also explored.

## Introduction

The projected rise of global population from the current 7 billion to over 9 billion within the next several decades [1,2] garners urgent needs for renewable energy and alternative foods. As global supplies of petroleum continue to decline, renewable nature fuels including biofuel are being explored. Biofuels are defined as the energy derived from raw biological materials, and show promise in harnessing adequate energy and reducing greenhouse gas emissions associated with fossil fuels. As corn and soybean are staple food crops for humans as well as the two main conventional feedstuffs that provide energy and protein for food-producing animals, the projected global population rise, along with the expansion of animal production, presents a serious threat to nutrition security for both humans and animals. However, the increasing use of these feedstocks for biodiesel and bioethanol production has driven up their global prices. From 2007 to 2011, the worldwide production of bioethanol nearly doubled from 49.6 to 84.6 billion liters [3]. In parallel, the price of corn was increased from $163 to $291 per metric ton over the four years [4]. In 2011, the United States produced approximately 12.4 billion bushels of corn, and 38% of which was used to produce bioethanol or to generate other co-products [3]. Apparently, current allocations of corn and(or) soybean for the biofuel and animal productions are unsustainable. Alternatives to these ingredients are required to maintain a harmonious infrastructure among the fuel, food, and feed industries.

Marine microalgae bear attractive properties for biofuel production [5-8]. Subsequently, the fat-extracted microalgal biomass derived from the biofuel production may be a promising carbon-neutral animal feed supplement [9-11]. In fact, our laboratory has recently demonstrated that the de-fatted biomass of *Staurospira sp*. with 19% crude protein replaced 7.5% of corn and soybean meal without affecting the growth performance or health status of broiler chickens [12]. Notably, the dual application of microalgae as a new source of biofuel and animal feed will help alleviate the greenhouse effects associated with current energy and food production.

## Microalgae for biofuel production

Marine microalgae sequester carbon dioxide (CO_2_) through photosynthesis, and may be used to produce biogas including methane and hydrogen via anaerobic processing [13-15]. While certain species of microalgae were recognized in the 1940s to yield high amounts of cellular lipids under selective growth pressures, it was not until the 1950’s when algae were viewed as a potential energy source, and were tested for methane gas production via anaerobic digestion of their cell carbohydrates [16,17]. The flexibility and(or) adaptivity of microalgal species to water and cultural conditions allows us to spare fresh water and arable land for crop production [18]. The land use efficiency of microalgae for biofuel production, grown with 30% oil content by weight, was 130 and 338 times greater than the conventional biodiesel feedstock soybean and corn, respectively [6].

While optimal growth conditions for microalgae are species-specific, photoautotrophic cultivation of these single cell species at large scales for biofuel and co-products depends on the technical and economic feasibility. At the present time, the photoautotrophic production of microalgae is marginally cost-effective only for generating value-added co-products or feed additives used in aquaculture [19,20]. In such productions, microalgae are grown in the presence of light within constructions such as open raceway ponds. To extract the lipids, microalgae are first de-watered. The concentrated biomass is subsequently processed to optimize the solvent extraction through cell disruption, particle size reduction, and drying [21]. The remaining microalgae skeleton after lipid extraction is the so-called de-fatted microalgal biomass to be used as an animal feed. Without the presumed feed application, the commercial microalgae cultivation and processing for biofuel production [22,23] remains largely cost-ineffective. Therefore, the feed application of the de-fatted biomass would not only create a new source of animal feed to mitigate the current competition with human food supply, but also help make the biofuel production of microalgae economically feasible.

## Microalgae for animal nutrition

### Feasibility of microalgae as animal feed

Although the use of whole microalgae in animal diets has long been studied, dating as far back as in the 1950’s, only recent literature has shown attempts to supplement lipid-extracted microalgae in animal diets [12,24]. Initially, researchers sought out methods to culture algae in ponds, and the developments of which were quickly followed by studies on algae supplementation into animal diets as a protein source [25,26]. Different sources of cultivated algae were effective in maintaining animal growth performance, and in some cases improving daily body weight gain. Sewage-grown *Chlorella* and *Scenedesmus* sp. gained attention as potential nutrient sources due to their high crude protein and carotenoid contents [27-30]. In 1952, Combs reported that supplementing 10% *Chlorella* sp. into a diet deficient in riboflavin and vitamin A improved feed efficiency and growth of chicks [27]. Later work showed no adverse response of growth by chicks to diets containing 20% sewage-grown, aluminum-free *Chlorella* and *Scenedesmus* sp., compared with those fed a corn-soybean meal based diet [28].

During the following decades, pond and tap water-grown algae were found to sustain fish growth in aquaculture, and 6-10% sewage-grown algae incorporated in a barley-based diet for growing-finishing pigs maintained their growth rate and feed conversion efficiency [31,32]. Other algal variants, such as the blue-green algae *Spirulina* sp., were also investigated not only for their effects on overall growth performance, but also on organ health and reproductive characteristics of animals. Ross and Dominy supplemented diets for broiler chickens with up to 20% blue-green algae, and found the 3 wk-old broilers experienced depressed growth when algae inclusion levels were higher than 10% [33]. At the inclusion rate of 12%, broilers showed slower growth in comparison with those fed 0, 1.5, 3.0, or 6.0% algae. Toyomizu et al. [34] fed broilers with up to 8% *Spirulina* sp. and reported no differences in their body weight, liver weight, abdominal fat, or kidney weight at 16 d of age. When early-weaned piglets were fed a diet with *S. maxima* replacing up to 12% of the total protein from skim milk, apparent digestibilities of the diets were reduced, but the piglet growth remained insignificantly different from that of the control pigs [35]. Fevrier and Seve [36] supplemented *S. maxima* into the diets of sows, and showed that the algae-fed sows had lower net weight gains with more and heavier piglets at the first reproductive cycle. By the end of the second reproductive cycle, the growth and litter characteristics were similar between the algae-fed and control groups, although the algae-fed sows produced a higher culling rate of piglets. Another study showed that feeding weanling pigs with a mixture of *S. maxima* and *A. platensis*, or *Chlorella* sp. in replacing 33% of soybean meal did not affect their body weight gain, feed efficiency, or incidence of diarrhea compared with pigs fed a control diet. Apparent toxicity or gastro-intestinal lesion was absent in the algae-fed pigs [35].

In contrast, the de-fatted biomass of microalgal species, derived from the biofuel production research, has only recently been shown feasibility in replacing corn and soybean meal in diets for poultry, swine, and cattle. Studies supplementing the de-fatted biomass from *Staurospira* sp. to replace 7.5% corn and soybean meal in diets for weanling pigs did not affect their overall growth performance or plasma biochemical indicators [37,38]. However, the pigs were incapable of tolerating a 15% replacement of corn and soybean meal [38]. Factors causing this intolerance might include amino acid imbalances, disruption of the acid:base balance, high ash content of the algal biomass, and(or) an overall reduction in the buffering capacity within the gastrointestinal tract of the weanling pigs. Austic et al. [12] replaced 7.5% of soybean meal with the de-fatted biomass of the *Staurosira* sp. in the diets of broilers, and showed decreased body weight gain and feed efficiency during the first three weeks of experiment. During the following three weeks, these differences were no longer seen. In the same study, broiler chicks fed a diet containing essential amino acids (Met, Lys, Ile, Thr, Trp, and Val) co-supplemented with 7.5% of the de-fatted biomass did not show growth performance differences from the control group. A diet with 10% lipid-extracted *Nannochloropsis oculata* meal was well tolerated by adolescent male rabbits, which showed similar final body weight, serum urea nitrogen, blood glucose, and organ histology in comparison with rabbits fed a control diet [39,40]. Finishing wethers maintained similar growth performance and carcass characteristics (*longissimus* muscle area, dressing percentage, marbling score, hot carcass weight, and subcutaneous adipose depth) when fed up to 20% de-fatted algal biomass on a dry matter basis as a protein replacement, in comparison with wethers fed a control diet [24]. In a study that examined effects of supplementing dietary dry matter with de-fatted *Lithothamnium calcareum* meal in Holstein cows, up to 1% algal meal in the diet mediated venous acid–base balance after acidosis induction, but did not improve the tract digestibility or growth performance of the cows [41].

### Nutritional values of microalgae

While the nutritional profiles of microalgae vary considerably with the species used, a large majority are characterized by protein, carbohydrate, and lipid contents that are comparable, if not superior, to conventional feedstuffs (Table 1). Dietary soybean meal typically contains up to 48% crude protein, and lays claim to a relatively well balanced amino acid profile. The diversity of microalgae makes certain species amenable to cultivation for diet-specific needs of humans and animals. A commonly cultivated algae species for human consumption: *S. maxima*, contains high levels of vitamin B_1_, vitamin B_2_, and β-carotene, and up to 71% crude protein with sufficient concentrations of all essential amino acids except for the sulfur-containing ones (Table 2) [5,42]. Since protein is considered to be the most expensive nutrient in animal feed [43], developing natural alternatives to soybean meal may be cost competitive.

**Table 1:** **Nutrient composition of conventional feedstuffs and various algae (% dry matter)**^**a**^

**Source**	**Crude protein**	**Carbohydrates**	**Lipids**
Soybean	37	30	20
Corn	10	85	4
Wheat	14	84	2
*Anabaena cylindrical*	43-56	25-30	4-7
*Arthrospira maxima*	60-71	13-16	6-7
*Chlorella vulgaris*	51-58	12-17	14-22
*Spirogyra sp.*	6-20	33-64	11-21
*Synechococcus sp.*	73	15	11

^a^Adapted from [11,44-46].

**Table 2:** **Amino acid profile of conventional protein sources and various algae (g/100 protein)**^**a**^

**Source**	**Ala**	**Arg**	**Asp**	**Cys**	**Glu**	**Gly**	**His**	**Ile**	**Leu**	**Lys**	**Met**	**Phe**	**Pro**	**Ser**	**Thr**	**Try**	**Tyr**	**Val**
Egg	-	6.2	11.0	2.3	12.6	4.2	2.4	6.6	8.8	5.3	3.2	5.8	4.2	6.9	5.0	1.7	4.2	7.2
Soybean	5.0	7.4	1.3	1.9	19.0	4.5	2.6	5.3	7.7	6.4	1.3	5.0	5.3	5.8	4.0	1.4	3.7	5.3
Dried Whole Milk	0.9	0.9	2.0	0.2	5.5	0.5	0.7	1.6	2.6	2.1	0.6	1.3	2.5	1.4	1.2	0.4	1.2	1.7
*C. vulgaris*	9.4	6.9	9.3	-	13.7	6.3	2.0	3.2	9.5	6.4	1.3	5.5	5.0	5.8	5.3	-	2.8	7.0
*D. bardawil*	7.3	7.3	10.4	1.2	12.7	5.5	1.8	4.2	11.0	7.0	2.3	5.8	3.3	4.6	5.4	0.7	3.7	5.8
*S. platensis*	9.5	7.3	11.8	0.9	10.3	5.7	2.2	6.7	9.8	4.8	2.5	5.3	4.2	5.1	6.2	0.3	5.3	7.1
*Aphanizomenon flos-aquae*	4.7	3.8	4.7	0.2	7.8	2.9	0.9	2.9	5.2	3.2	0.7	2.5	2.9	2.9	3.3	0.7	-	3.2

^a^Based on reference [11].

Among all dietary amino acids, lysine and methionine are the first and second limiting amino acids. Many microalgal species contain relatively high amounts of lysine, but as previously described, are somewhat deficient in the sulfur-containing amino acids: cysteine and methionine [5]. To maximize amino acid utilization by animals, diets are typically formulated by mixing different feedstuffs to balance amino acid profiles and(or) by supplementing synthetic amino acids to meet their nutrient requirements. Austic et al. [12] reported that the decreased growth performance of broilers fed the de-fatted *Staurospira* sp. biomass in the first three weeks was prevented by the supplementation of essential amino acids. In a laying hen study, a 7.5% replacement of corn and soybean meal with the de-fatted microalgal biomass did not negatively affect hen production or health parameters, yet significantly increased the redness and decreased both the lightness and yellowness of the egg yolks [47]. However, inclusions of the biomass at 10% reduced feed intake, plasma uric acid, and egg albumen weight. Up to a third of soybean meal was successfully replaced in the diets of weanling pigs by algae biomass from the cyanobacteria *S. maxima*, *Arthrospira platensis*, and *Chlorella* sp. [35]. In another study conducted on laying hens, an inclusion of 10% *Porphyridium* sp. red algal biomass did not affect their body weight, egg production rate, or egg weight, but lowered egg yolk cholesterol levels by 24% [48].

## Microalgae for value-added animal products

The research on microalgal biomass supplementation in food-producing animals has opened a new gateway to improve human health. A study conducted in the last decade showed not only 10% enhancement of growth but also potential in producing iodine-rich pork for human consumption when pigs were fed algae containing naturally high iodine [49]. Likewise, microalgae may be cultivated for their advantageous fatty acid profile (Table 3), with notable enrichment in omega-3 (n-3) polyunsaturated fatty acids (PUFA) including arachidonic acid, docohexaenoic acid (DHA), eicosapentaenoic acid (EPA), and γ–linoleic acid [21]. The enriched concentrations of these omega-3 fatty acids by a variety of microalgal species represent a largely untapped natural resource with multiple health implications for both humans and animals. Among many fat sources, fish products represent the major source of n-3 fatty acids [50]. However, marine fish species are incapable of synthesizing n-3 fatty acids by themselves; they may obtain n-3 fatty acids by consuming microalgae or other algae-consuming fish [51]. Most notably, dietary intakes of n-3 fatty acids are associated with decreased risks of chronic diseases [52]. However, the average fish consumption in the U.S. has not changed since 1983, at approximately 6.5 kg per year [53]. To increase the public consumption of n-3 fatty acids, studies have been conducted with full-fatted or de-fatted microalgae to generate n-3 fatty acid-fortified animal products such as milk, meats, and eggs. Apparently, the profile of PUFA in the de-fatted microalgal biomass is affected by the lipid extraction procedure. While a practical solvent extraction can remove 95% of lipids from oilseeds, it is ineffective to extract microalgal lipid due to the relatively high moisture content and cellular elasticity [21]. Grima et al. [54] reported that the lipid extraction yields of dried algal biomass range from 50 to 93% depending on the polarity of solvent systems. In fact, a higher proportion of long chain polyunsaturated fatty acids in the de-fatted biomass may bolster its appeal as an n-3 fatty acids source for developing value-added animal products.

**Table 3:** **Generalized fatty acid profiles (%) of oil extracts from** ***Spirulina maxima*** **(SP),** ***Chlorella vulgaris*** **(Cv),** ***Scenedesmus obliquus*** **(Sc),** ***Dunaliella tertiolecta*** **(Dt),** ***Nannochloropsis*** **sp, (Nanno), and** ***Neochloris oleabundans*** **(Neo)**^**a**^

**Fatty acid**	**Sp**	**Cv**	**Sc**	**Dt**	**Nanno**	**Neo**
14:0	0.34	3.07	1.48	0.47	7.16	0.43
16:0	40.16	25.07	21.78	17.70	23.35	19.35
16:1	9.19	5.25	5.95	0.88	26.87	1.85
16:2	N.D.	N.D.	3.96	3.03	0.39	1.74
16:3	0.42	1.27	0.68	1.24	0.48	0.96
16:4	0.16	4.06	0.43	10.56	N.D.	7.24
18:0	1.18	0.63	0.45	N.D.	0.45	0.98
18:1	5.43	12.64	17.93	4.87	13.20	20.29
18:2	17.89	7.19	21.74	12.37	1.21	12.99
18:3	18.32	19.05	3.76	30.19	N.D.	17.43
18:4	0.08	N.D.	0.21	N.D.	N.D.	2.10
20:0	0.06	0.09	N.D.	N.D.	N.D.	N.D.
20:1	N.D.	0.93	N.D.	N.D.	N.D.	N.D.
20:2	0.48	N.D.	N.D.	N.D.	N.D.	N.D.
20:3	N.D.	0.83	N.D.	N.D.	N.D.	N.D.
20:4	N.D.	0.23	N.D.	N.D.	2.74	N.D.
20:5	N.D.	0.46	N.D.	N.D.	14.31	N.D.
SFA	41.74	28.86	23.71	18.17	30.96	20.76
MUFA	14.62	18.82	23.88	5.75	40.07	22.14
PUFA	37.35	33.09	30.78	57.39	19.13	42.46

^a^Based on reference [55].

Microalgal biomass or oil may be supplied in the feed of ruminants to manipulate their milk fatty acid composition. The content of n-3 fatty acids in milk, especially DHA, has been shown to increase with the inclusion of algal biomass or oil, without affecting the milk yield [56-58]. However, a study with similar dietary algae incorporation reported a decrease in milk fat content [59]. Many studies in ruminants have focused on producing n-3 fatty acid-fortified milk. In such an attempt, Stamey et al. [51] supplied either algal biomass or oil to dairy cows. The overall milk fat and yield were not affected by either supplement, but the milk DHA (C22:6) content was increased by both supplementations. Glover et al. [57] reported that feeding microalgae and fresh forage decreased total milk fat content, but elevated its DHA concentration. A parallel study was conducted with sheep to determine if algae and (or) their co-supplementation with sunflower oil in the diet could enhance the nutrient profile of milk [59]. While the milk yield was unaffected by the dietary treatments, the milk fat content was decreased and the milk DHA concentration was increased as dietary algae concentration rose. Franklin et al. [58] determined that feeding dairy cows with either algae protected against ruminal biohydrogenation or unprotected algae elevated the milk DHA concentration without affecting the milk fat content. Collectively, the supplementation of microalgae to the diets of ruminants increased the concentration of n-3 PUFA in milk, with a mixed effect on the milk fat content.

Likewise, fatty acid compositions in meats and eggs are also affected by dietary supplementations of microalgae. In lambs and horses, dietary microalgae increased the n-3 fatty acid content in meat and blood, respectively [60,61]. In pigs, dietary microalgae increased DHA concentrations in the loin and subcutaneous fat [62]. However, this increment did not manifest as a dose-dependent response [63]. To enrich eggs with n-3 fatty acids, flaxseed is usually supplemented in diets of laying hens [64]. Alternatively, dietary algae have been shown to increase n-3 and decrease n-6 fatty acids in eggs [64,65]. Supplementing flaxseed at 10% or higher usually decreases egg production and the body weight of hens. Moreover, such high flaxseed supplementations may induce fatty acid oxidation, resulting in off-odor eggs [66]. Herber and Van Elswyk [65] supplemented up to 4.8% of golden marine algae into the diet of hens, and reported increased levels of DHA with a concomitant reduction of n-6 fatty acids. The inclusion of algae in the diet affected neither the egg production nor egg quality in the 56 wk-old hens. When up to 4.3% of fermented *Schizochytrum* sp. was included in the diets of hens, their egg production and feed conversion ratio were elevated [67]. The 0.86% and 4.3% supplementation of the microalgae elevated the DHA content to 134 and 220 mg/egg, respectively.

## Special considerations of microalgae

Despite their high nutritional values and health implications, caution must be taken to avoid possible toxicity of the microalgal biomass [8]. Among over 200,000 existing algal species, approximately 35,000 species are characterized [68] without apparent toxicological property [5]. However, certain species contain biogenic toxins including purines and non-biogenic toxins such as heavy metals. Some algal species are known to rapidly accumulate heavy metals at concentrations higher than their surroundings [5], while others generate pathological metabolites that cause neuro-degenerative disorders [69]. Such species require establishment and evaluation prior to their commercialization as feed supplements. Currently, no official regulations for heavy metals in microalgal products exist [5].

In spite of the relatively high crude protein contents, many microalgae species may still show limited biological values for the proteinaceous biomass due to the presence of non-protein nitrogen that consists of nucleic acids, nitrogen-containing cell walls, and amines. As single cell proteins, microalgae are photosynthetic unicellular eukaryotes containing nucleic acids that may represent approximately 10% of the crude protein [5]. Ruminal bacteria are effective in utilizing non-protein nitrogen to synthesize protein [70]. Simple-stomached animals lack ruminal microbes and thus are incapable of utilizing the relatively high levels of non-protein nitrogen in the microalgal biomass. Because the individual amino acid content and digestibility are important factors for formulating diets to support the maximum growth of animals without excreting excess nitrogen, it is necessary to optimize dietary amino acid profile and digestibility when the microalgal biomass is used to replace conventional feedstuffs.

Notably, the microalgal cell wall is largely indigestible by simple-stomached animals, yet contains immunostimulating properties that may provide added value to the biomass [71,72]. Approaches to improve the absorption of microalgal nutrients include employing enzymatic treatments to enhance cell wall digestibility [71], as well as developing genetically-altered microalgae that lack cell walls [73]. Microalgal solubility is high at alkaline conditions [74]. Comprising approximately 10% cellular dry matter [71], the cell wall of *Chlorella* sp. contains high levels of β-1,3-glucan, a bio-active immunostimulating free-radical scavenger [75]. Other species, such as those of cyanobacterial origin, have well-recognized anti-tumor, anti-bacterial, and anti-viral properties [76]. Microalgal cell wall-derived sulfated polysaccharides and carrageenans show strong T and B cell mitogenic effects [77]. Animals fed the whole *Spirulina* sp. samples accumulated greater degrees of cytotoxic lymphocytes that were critical to innate immunity, and had elevated phagocytic activity and antibody production [78].

## Conclusions

The use of microalgal biomass in animal feed will not only improve human and animal food security, but also facilitate cost-effective biofuel production and reduces greenhouse gas production of agriculture [6,79-81]. Recent estimates indicate that 30% of the global algal production is used by the animal feed industry [5], amounting to a fast-growing $300 million in retail value [82]. Pragmatic species-specific large-scale algae refinery techniques must be devised to reduce the cost of the biomass production. It is necessary to determine limiting factors of the microalgal biomass that hinder its digestion and utilization by animals. Novel technology should be explored to improve microalgal nutrient utilization, and their long term nutritional and metabolic effects should be assessed. In addition, the production of DHA from microalgal biomass alone is a rapidly growing market. The last estimate is that approximately 300 tons is produced to generate $1.5 billion market value each year [72,82]. However, the tremendous potential of using the microalgal biomass in producing DHA/EPA enriched eggs, meats, and milk for improving human health is yet to be fully explored.

## Abbreviations

DHA: Docosahexaenoic acid; EPA: Eicosapentaenoic acid; PUFA: Poly unsaturated fatty acid; SFA: Saturated fatty acid.

## Competing interests

The authors declare that they have no competing interests.

## Authors’ contributions

KL, JK, and XGL wrote the review. All authors read and approved the final manuscript.
